# Reversible electrical switching of nanostructural color pixels

**DOI:** 10.1515/nanoph-2022-0646

**Published:** 2023-01-17

**Authors:** Shutao Zhang, Jun Zhang, Wei Peng Goh, Yan Liu, Febiana Tjiptoharsono, Henry Yit Loong Lee, Changyun Jiang, Jun Ding, Joel K. W. Yang, Zhaogang Dong

**Affiliations:** Institute of Materials Research and Engineering, A*STAR (Agency for Science, Technology and Research), 2 Fusionopolis Way, #08-03 Innovis, 138634, Singapore, Singapore; Singapore University of Technology and Design, 8 Somapah Road, 487372, Singapore, Singapore; Department of Materials Science and Engineering, National University of Singapore, 9 Engineering Drive 1, 117576, Singapore, Singapore

**Keywords:** Mie resonance, reversible electrodeposition mirror, silicon nanostructures, tunable high-resolution color display

## Abstract

Electrical switching of nanophotonic structural color elements is a promising approach towards addressable color switching pixels for next generation reflective displays. However, electrical switching between the primary colors to colorless near-white state remains a challenge. Here, we present a reversible electrical switching approach, relying on the electrocoagulation of Ag nanoparticles between silicon nanostructures that support Mie resonances. The electrodeposited Ag nanoparticles enable the excitation of the hybrid plasmon-Mie resonance as supported on Ag-silicon nanostructures, resulting in a large spectral transformation. Importantly, this process is reversible. This device design outperforms other designs in terms of electrotonic color control since it is highly stable and reliable for use in high-resolution reflective displays, such as colored electronic papers and smart display glass, where the combination is scalable to other nanostructure designs and electrolytic solutions.

## 1 Introduction

Nanostructures supporting localized plasmon resonances [1, 2] or Mie resonances in general [3–6] enable color prints at unprecedented resolutions [7–10] often beyond the diffraction limit of visible light microscopy. This color printing technology provides the platform for next-generation reflective (micro)displays with low energy consumption when structural resonances can be tuned. Reversible switching or tuning of these localized optical resonances is necessary to realize display functionality [11, 12]. So far, various tuning approaches have been explored in the literature, such as chemical reactions [13], thermal annealing process [6, 14], [15], [16], phase transition [17–20], mechanical stress [21–23], temperature change [24–26], hydrolysis [27], optical excitation [28], and electrical voltage [29–44].

Among all these tuning mechanisms, electrical voltage tuning is arguably the most promising one towards practical applications. Electrodeposition is one electrical tuning approach that offers the ability to reversibly alter the geometry of nanostructures. For instance, the use of a combination of bimetallic deposition and electrochemical bias can achieve plasmonic modulation of color change through the deposition of Ag particles into Au holes [29]. In addition, metal plasmonic nanostructures with different curvatures are used in combination with the conductive polymer LiClO_4_ to achieve high speed switching between structural colors and its dark states [31]. Similarly, electrochemical deposition of the copper onto dielectric grating slits was shown to change its modal interference for color tuning [32]. Similar studies have been also carried out using electrochromic polymers being coated on the slit sidewalls to modify the interactions between surface iso-excitations to achieve fast response and high contrast color switching [33]. However, among these investigations, electrical switching between the primary colors (*i.e.*, red, green, and blue) and its colorless near-white state still remains a challenge.

In this paper, we design and fabricate an electrical switching display element based on silicon nanodisks with Mie resonances, which are integrated with an electrochemical material [45, 46]. The display device consists of silicon nanodisks with tin-doped indium oxide (ITO) as the bottom electrode and glass with transparent ITO on the surface as the top electrode, where dimethyl sulfoxide (DMSO) electrolyte solution containing silver nitrate [47, 48] is used to deposit Ag film to realize color tunablity. The white light source is incident onto the silicon nanodisks through the glass substrate, and the structured color is generated due to the size-dependent Mie resonances of silicon nanodisk arrays. When a negative voltage (1–3 V) is applied, Ag ions at the surface of ITO are reduced to form Ag nanostructures that are deposited into the region between the silicon nanodisks, changing the reflection of the incident light to realize the dynamic tuning of colors. The device can be applied to achieve the electrically tunable reflective display devices, such as color electronic papers, where the color pixels will go back to the original states after the voltage is removed or applying a reverse voltage.

## 2 Results and discussion

### 2.1 Design schematic

**Figure 1:**
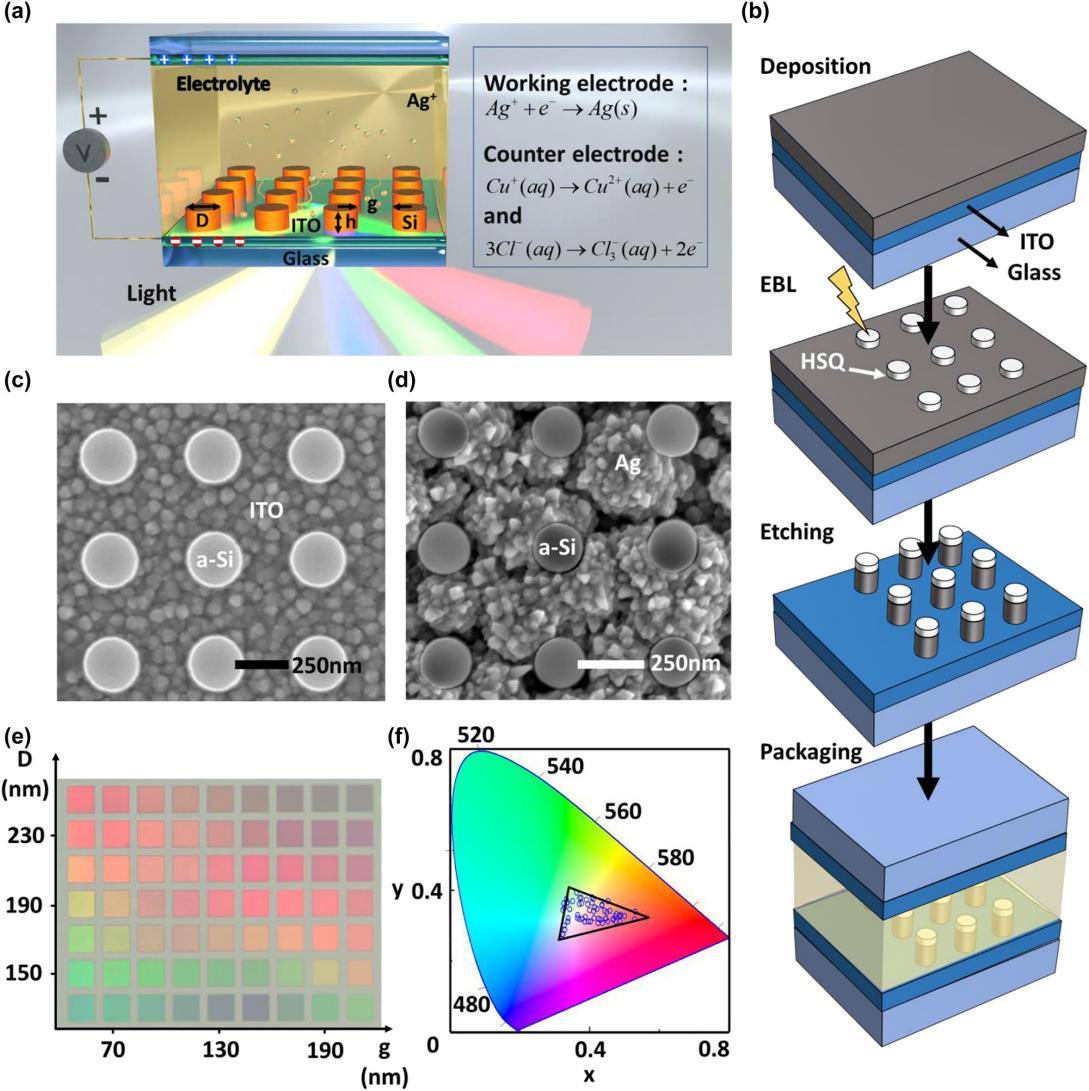
Design, fabrication process, and characterization of the high-resolution color pixels with electrical switching capability via electrochromic reaction. (a) Schematic of the device consisting of the bottom electrode, the top electrode, and the electrolyte. The bottom electrode is a 25-nm-thick ITO on glass substrate with amorphous silicon (a-Si) nanodisks. The top electrode is a 180-nm-thick ITO on glass substrate, where the electrolyte is a DMSO solution containing AgNO_3_, CuCl_2,_ and LiCl. The diameter, gap size, and height of the nanodisk arrays are denoted as *D*, *g*, and *h*, respectively. (b) Fabrication process of a-Si nanostructures (see details in the methods section). (c)–(d) Scanning electron microscope (SEM) images of the a-Si nanodisks before and after Ag electrodeposition (applying 2 V for ∼5 s). (e) Bright-field optical micrographs of the device after encapsulation. (f) CIE 1931 chromaticity diagram of the color pixels based on the measured reflectance spectra.

Figure 1(a) presents the design of the electrically tunable display element, which includes three parts, *i.e.*, top electrode, bottom electrode, and DMSO electrolyte containing AgNO_3_, CuCl_2,_ and LiCl. The top electrode is a glass with 180-nm-thick ITO film. The bottom electrode is a glass substrate with 25-nm-thick ITO, consisting of nanostructures with a fixed height (*h*) of 100 nm, diameter (*D*) of 130–250 nm with an incremental step of 20 nm and gap (*g*) of 50–230 nm with an incremental step of 20 nm, where the cavity formed between the top and bottom electrodes is 400 µm. When a negative voltage is applied on the bottom electrode, the following chemical reactions (Eqs. (1)–(3)) [47, 49] occur.

The reaction on the bottom (working) electrode is:

(1) $${Ag}^{+}+e^{-}\to Ag\left(s\right)$$

The reaction on the top (counter) electrode is:

(2) $${Cu}^{+}\left(aq\right)\to {Cu}^{2+}\left(aq\right)+e^{-}.$$

And

(3) $$3{Cl}^{-}\left(aq\right)\to {Cl}_{3}^{-}\left(aq\right)+2e^{-}.$$

The negatively biased bottom electrode with Si nanodisks provides electrons for AgNO_3_ to produce Ag nanoparticles, which will be grown around amorphous Si nanodisks. Then, the Mie resonance of Si nanodisks disappears and the reflectance of bottom electrode is largely enhanced due to Ag film. When reverse the bias, with a positive voltage (∼0.5 V) applied on the bottom electrode, the reverse reaction occurs, and the Ag film is dissolved into the transparent electrolyte. Therefore, the Mie resonance of Si nanodisks is recovered, and the pre-designed structure color is restored accordingly. Even upon removal of the applied voltage with an open external circuit, a self-bleaching process (Eq. (4)) [47, 49] will also occur to dissolve the Ag film.

The self-bleaching reaction on the bottom electrode is:

(4) $$Ag+{Cu}^{2+}\to {Ag}^{+}+{Cu}^{+}.$$

The schematic for illustrating the fabrication process is shown in Figure 1(b). First, the fabrication started from the deposition of 100-nm-thick amorphous Si thin film onto the glass substrate with a conductive 25-nm-thick ITO film, by using plasma-enhanced chemical vapor deposition (PECVD). Then, electron beam lithography (EBL) was carried out after, where 30-nm hydrogen silsesquioxane (HSQ) resist (Dow Corning, XR-1541-002) was spin-coated onto the sample surface. After the electron beam lithography, the HSQ resist is developed by using the NaOH/NaCl salty solution [50]. After etching process with ICP reactive ion etching, Si nanodisks are formed on the top surface of bottom electrode. The device is obtained by assembling and encapsulating the bottom and top electrodes and filling the cavity with electrolyte (DMSO with AgNO_3_, CuCl_2,_ and LiCl). The detailed fabrication steps and processes are given in the Methods section.

Figure 1(c) shows a scanning electron microscope (SEM) image of silicon nanodisks on ITO glass, and the surrounding small grains are due to the ITO film. Figure 1(d) shows the SEM image of silicon nanodisks with the deposited Ag nanostructures. These Ag nanostructures around the Si nanodisks not only provide high reflectance, but also the hybrid Si–Ag nanostructures support a hybrid plasmon-Mie resonance. Therefore, this deposition of Ag nanostructures will lead to the near-white colorless state. The bright-field optical microscope image of the typical pixels is shown in Figure 1(e). To characterize the wavelength-dependent properties of the Si nanodisks, the nanostructured Si arrays with different diameter (*D*) and gap size (*g*) were prepared, where *D* and *g* values increase from 130–250 nm and 50–230 nm, respectively, with an incremental step of 20 nm. The size of each color patch pixel is 10 × 10 μm^2^. Subsequently, a CIE1931 chromaticity is obtained based on the measured reflectance spectra, as shown in Figure 1(f).

### 2.2 Electrically switching test

**Figure 2:**
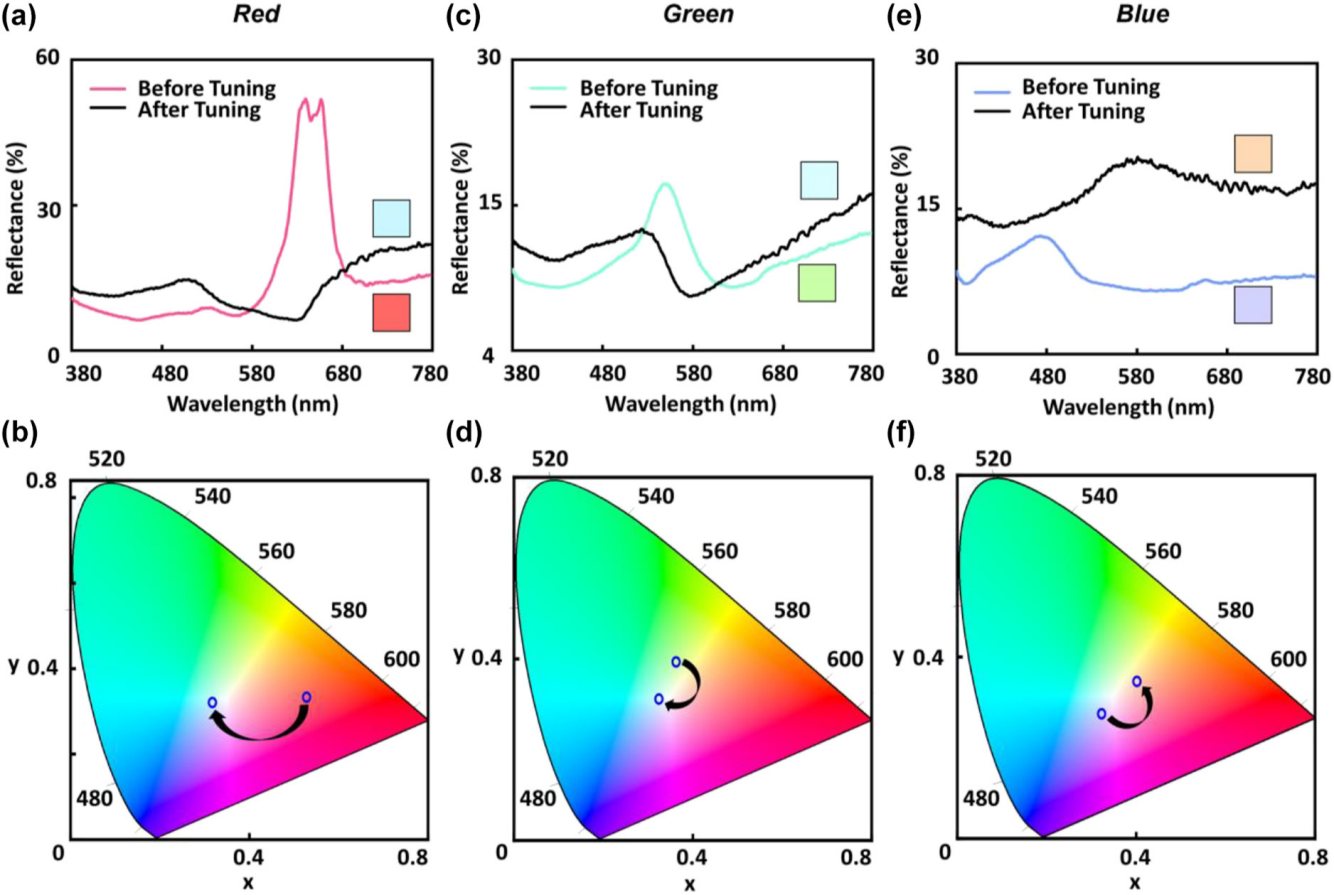
Electrical switching process of the respective red, green, and blue color palettes. (a) Reflectance spectrum of red pixel (Si nanodisk with *D* = 250 nm and *g* = 50 nm) before and after switching. (b) Switching trace of the red pixel in CIE 1931 chromaticity coordinate map. (c) Reflectance spectrum of green pixel (Si nanodisk with *D* = 170 nm and *g* = 50 nm) before and after switching. (d) Switching trace of green pixel in CIE 1931 chromaticity coordinate map. (e) Reflectance spectrum of blue pixel (Si nanodisk with *D* = 130 nm and *g* = 110 nm) before and after switching. (f) Switching trace of blue pixel in CIE 1931 chromaticity coordinate map.

After fabricating the device, a bias voltage of 1–3 V is applied (*i.e.*, the bottom electrode is connected to the negative end of the bias voltage). The thickness of the Ag nanostructures is tuned by controlling the amplitude of bias voltage and reaction time. The reflectance spectra of red pixel (*D* = 250 nm and *g* = 50 nm), green pixel (*D* = 170 nm and *g* = 50 nm) and blue pixel (*D* = 130 nm and *g* = 110 nm), before and after color switching, are shown in Figure 2(a), (c) and (e), respectively. The inserted figures are the related color pixels before and after switching. Here, the DC bias voltage of 2 V is used. Before switching, the red, green and blue pixels show reflection peaks at around 640, 550, and 480 nm, respectively. After growing the Ag nanostructures, it will enable the excitation of the hybrid plasmon-Mie resonance as supported on Si–Ag nanodisk, where the similar hybrid plasmon-Mie resonance has been reported recently for the miniaturized color detectors [51]. The switching trace in the CIE 1931 chromaticity diagram of red, green, and blue pixels before and after switching are shown in Figure 2(b), (d), and (f), respectively. The pixels eventually converge to the colorless near-white state after applying the voltage, resulting in a change from initial primary colors with high transparency to pure white opaque reflective state.

**Figure 3:**
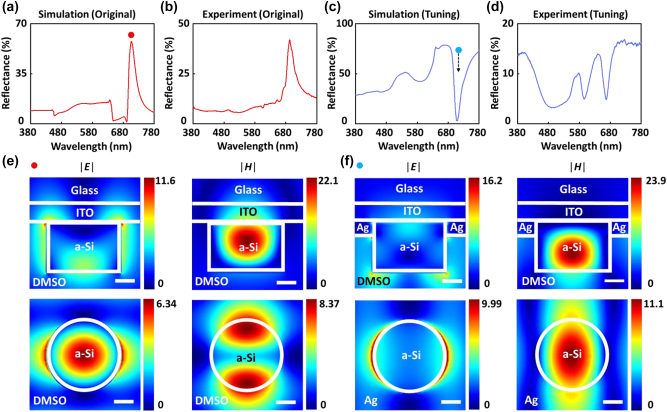
Finite-difference time-domain (FDTD) simulation analysis on the resonant modes as supported on the color pixel (*D* = 200 nm and *g* = 170 nm) before and after switching. (a)–(b) Simulated and measured reflectance spectra before color switching. (c)–(d) Simulated and measured reflectance spectra after color switching. (e) Simulated spatial distribution of electrical field magnitude (| ***E*** |) and magnetic field magnitude (| ***H*** |) before color switching at the peak wavelength of 710 nm. (f) Simulated spatial distribution of electrical field magnitude (| ***E*** |) and magnetic field magnitude (| ***H*** |) after color switching at the dip wavelength of 710 nm. (e)–(f) the upper panels show the cross-section view in *X*–*Z* plane and the lower panels show the top view at *Z* = 130 nm in *X*–*Y* plane. The scale bar denotes 50 nm.

To better understand the color switching mechanism of the silicon nanodisks, FDTD simulations were carried out to obtain the simulated reflectance in the wavelength range of 380–780 nm, as shown in Figure 3(a). There is a sharp peak at the wavelength of 690 nm, which is caused by strong Mie resonance of Si nanodisk. Figure 3(b) shows the measured reflectance for the pixel with the same geometric parameters. A sharp peak at the wavelength of 690 nm is also obtained, indicating that the experimental result is well agreed with simulation. With an idealized thin Ag film surrounding the Si nanodisk, the original peak at 690 nm becomes a dip, as shown in Figure 3(c) and (d). Therefore, the Mie resonance of Si nanodisk transits into more complex hybrid plasmon-Mie resonance modes with a strong absorption. Nevertheless, there exists some discrepancy between the simulated spectrum in Figure 3(d) with respect to the measured one as shown in Figure 3(c). The main reason is that the deposited Ag film is inhomogeneous as shown in the SEM image (Figure 1(d)), with the random Ag nanoparticle islands with random gaps and shapes, instead of being the flat Ag films. In the simulation, it is not easy to generate the same morphology model to consider all these randomness. To further observe the nature of the explained absorption peaks, the spatial distribution of the electrical field | ***E*** | and magnetic field | ***H*** | of the silicon nanodisk were simulated and the results are plotted in Figure 3(e) and (f). The Mie resonance of a-Si nanostructures can be seen clearly before switching in Figure 3(e). Meanwhile, after depositing Ag film, the hybrid plasmon-Mie resonance on Si–Ag is supported as shown in Figure 3(f), where the localized plasmon features exist at the interface between a-Si and Ag, which is commonly seen in the typical plasmonic systems with dielectric–metal interfaces [52, 53].

### 2.3 Reliability cycle test

**Figure 4:**
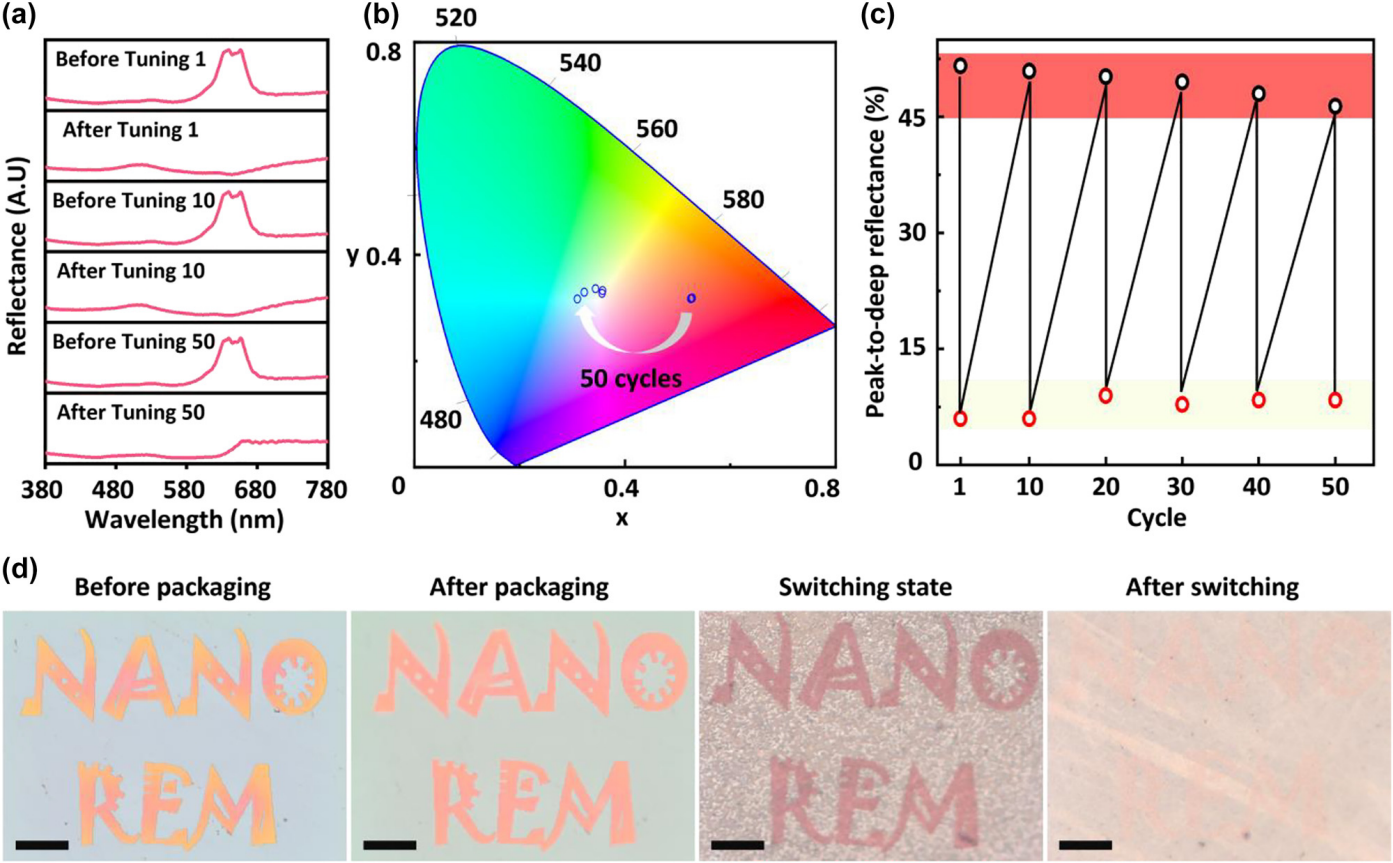
Cyclability test of the color pixel for Si nanostructure array with a diameter *D* of 250 nm and a gap size *g* of 50 nm. (a) Reflectance of pixels before and after switching for 1, 10, and 50 times. (b) Switching trace of pixel color in the CIE 1931 chromaticity coordinates for 50 cycles. (c) Peak-to-dip reflectance of pixel in 50 times cyclability test. (d) Demonstration of active color display: Optical microscope images of the “*NANO REM*” words in four states (before packaging, after packaging, switching state, and after switching) obtained from the red pixel design. The scale bar denotes 50 μm.

The cyclability performance is very important factor to characterize a tunable display device. The test was carried out with the red pixel (*D* = 250 nm and *g* = 50 nm) at a bias voltage of 2 V. The reflectance of red pixel before and after switching for the 1st, 10th, and 50th time is shown in Figure 4(a), respectively. After 50 cycles, the saturation of the color remains high and there is no significant peak reduction or shift, which shows the excellent cyclability and stability. The corresponding color plot in the CIE 1931 chromaticity coordinates is shown in Figure 4(b). It shows that the “red” state is quite stable (*i.e.*, there is almost no coordinate changing in the “red” state). However, due to differences in integration times during the spectral measurement process, the coordinate of colorless near-white state distributes in a small region. Because the work principle of electrically tuned color is that the Mie resonance of the original structure color is changed into the hybrid plasmon-Mie resonance after depositing Ag film, the stability of the device can be assessed by measuring the change in peak reflectance to notch in the Mie resonance region. Figure 4(c) plots the changes over 50 cycles. In the red and near-white regions, the device shows no significant change. Finally, the *“Nano REM”* image is created by using red pixels, where “*REM*” stands for reversible electrodeposition mirror. Figure 4(d) shows the optical microscope images before and after packaging, the switching state and the one after switching. It shows that after encapsulation, the sample had a more uniform and vibrant red color and it was able to quickly switch between “red” to “white” at a bias voltage of 2 V (see the supporting information for the switching video), with a typical switching time of ∼1 s.

**Video 1:** 

## 3 Conclusions

We presented an electrically tunable color display device, which is based on the electrodeposition of Ag film onto the silicon nanoantenna arrays with Mie resonances. With this design, we can switch between the primary color states and its colorless near-white states, in a reversible switching manner. At the same time, further improvements are needed to improve the color saturation with advanced nanophotonic designs, switching between the saturated colors and its black states, as well as exploring the mechanism to achieve the cyclic switching process for more than million times [54–56].

## 4 Methods

### 4.1 Fabrication

**Growth of amorphous-Si on ICP CVD**. Amorphous silicon (a-Si) was grown by using inductively coupled plasma chemical vapor deposition tool (ICP-Oxford Instruments Plasmalab Systems 100) with a DC power of 100 W, a coil power of 300 W, and a process pressure of 8.0 mTorr at the temperature of 250 °C. The gases used are SiH_4_ and Ar with flow rates of 48 sccm and 30 sccm, respectively. The time taken to grow 130 nm a-Si is about 7 min.

**Electron beam lithography (EBL) of Si Nanostructures**. A ∼30 nm etching mask (Hydrogen silsesquioxane (HSQ)) was created on the surface of Si (100 nm)/ITO (45 nm)/Glass sample (3 × 3 cm^2^) by using an EBL (Elionix ELS-7000). First, HSQ resists (Dow Corning XR-1541-002) were spin-coated onto a cleaned sample at 5000 round-per-minute (rpm) to obtain a HSQ thickness of ∼30 nm. Then, electron beam exposure was carried out with an electron acceleration voltage of 100 keV, beam current of 500 pA, and an exposure dose of ∼12 mC/cm^2^. After completing the exposure, the pattern was immediately developed by NaOH/NaCl salty solution (1% wt./4% wt. in de-ionized water) for one minute [57]. Then the sample was flushed with de-ionized water for one minute to stop the development. Finally, the sample was rinsed using isopropanol alcohol (IPA) and dried by a continuous flow of nitrogen gas.

**Inductively coupled plasma (ICP) for silicon etching**. The etching of a-Si was carried out by inductively coupled plasma reactive ion etching tool (ICP, Oxford Instruments Plasmalab System 100), with ICP power of 300 W and RF power of 100 W, using Cl_2_ gas, under a process pressure of 5 mTorr and a chiller temperature of 10 °C [58].

**Fabrication of the electrically switching color pixels**. AgNO_3_, CuCl_2_ and LiCl were obtained from Sigma Aldrich and used without further purification while anhydrous dimethyl sulfoxide (DMSO) was purchased from Kanto Chemicals. To prepare the electrolyte, 0.1 M of AgNO_3_, 0.05 M of CuCl_2,_ and 0.5 M of LiCl were mixed in DMSO. The contents were stirred overnight in an N_2_ environment on a hotplate at 60 °C. The fabrication of the reversible electrodeposition mirror (REM) device comprised 2 indium tin oxide (ITO) glass substrates separated by a polyimide spacer to form a 400 µm thick cavity. The electrolyte was then introduced into the cavity before sealing it up with Alteco 3-Ton Quick Epoxy Adhesive.

### 4.2 Characterizations

Scanning electron microscope images were taken at 5 keV accelerating voltage (SEM, Hitachi, SU8220). Optical microscopy images were taken by using an Olympus microscope (MX61) with a 20 × objective lens and an OLYMPUS SC30 camera, where the software “analySIS” was used to acquire the images with an exposure time of 25 ms. The illumination light source was a halogen lamp (U LH100 3, 100 W) with a linear polarizer (U-AN360−3). The optical reflectance spectra were measured by using a CRAIC UV−vis-NIR microspectrophotometer QDI 2010 (×5 objective lens, Zeiss A-plan with an NA of 0.12) with a broadband light source (75-W xenon lamp). Keithley 2450 source meter was used for voltage control, where the electrode having silicon nanoantennas was connected to the ‘Force-Lo’ of the source meter and the upper ITO glass electrode was connected to the ‘Force-Hi’ of the source meter. An output voltage between 1 and 3 V is output from the source meter, where a current limit of 10 mA is set for protecting the device.

### 4.3 Numerical simulations

Three-dimensional finite-difference time-domain (3D-FDTD) simulations were carried out to obtain the far-field spectra and the near-field electromagnetic field distributions in this nanostructure, using Lumerical FDTD Solutions software. The whole structure in Figure 1(a) has a film stack of glass/ITO/DMSO/a-Si/ITO/glass. The amorphous silicon (a-Si) nano disk was illuminated with a white light source (plane wave) at the backside of the glass with a normal incident condition. The periodic boundary condition has been used along *x*- and *y*-directions to mimic the periodic structure. Perfectly matched layers (PMLs) are employed on top and bottom of the structure along the *z*-direction to absorb the outgoing wave. In the simulation region, the mesh size was set to be 2 nm × 2 nm × 2 nm. The a-Si nanodisk with a diameter of 200 nm, the height of 100 nm on 25 nm ITO/glass substrate, which immersed into DMSO_AgNO_3_ chemical solution with a refractive index of 2.48. The monitor was placed above the source to record the reflectance spectrum. The material properties of a-Si were chosen based on the optical dielectric constant taken from previous ellipsometry measurements. The electric field and magnetic field distributions were collected, as well as the reflectance spectra.
